# Unravelling the complex interplay of age, comorbidities, and multimorbidities in COVID-19 disease progression: Clinical implications and future perspectives

**DOI:** 10.1016/j.heliyon.2024.e35570

**Published:** 2024-08-02

**Authors:** Maria Shoukat, Haseeb Khan, Wajid Munir, Moona Nazish, Abdulwahed Fahad Alrefaei, Mohammed Fahad Albeshr, Anwar Ali, Saad Ahmed, Afsheen Mansoor, Massab Umair, Muhammad Suleman Rana, Malik Badshah

**Affiliations:** a Department of Microbiology, Faculty of Biological Sciences, Quaid-i-Azam University, Islamabad, Pakistan; b Isolation Hospital and Infections Treatment Center, Islamabad, Pakistan; c Department of Plant Sciences, Quaid-i-Azam University, Islamabad, Pakistan; d Department of Zoology, College of Science, King Saud University, P.O. Box 2455, Riyadh, 11451, Saudi Arabia; e University Hospital Kerry, Ritass, Tralee, Co. Kerry, Ireland; f Saidu Group of Teaching Hospitals, Swat, Pakistan; g Department of Dental Material Sciences, School of Dentistry, Shaheed Zulfiqar Ali Bhutto Medical University, Islamabad, Pakistan; h National Institute of Health, Islamabad, Pakistan

**Keywords:** COVID-19, Comorbidity, Multimorbidity, Age, Hypertension, Diabetes mellitus, Chronic kidney disease, Ischemic heart disease

## Abstract

**Introduction:**

The COVID-19 infection as an inflammatory disease has posed significant challenges to global public health due to multi-factor risks associated with it leading to disease severity and mortality. Understanding the effect of age and comorbidities on overall disease progression is crucial to identify highly susceptible individuals and to develop effective disease management strategies in a resource limited country like Pakistan.

**Methodology:**

A retrospective study was conducted on hospitalized COVID-19 patients to assess the prevalence of various comorbidities among different age groups and their effect on disease severity and mortality rate.

**Results:**

In this retrospective study, a cohort of 618 hospitalized COVID-19 patients was analyzed, consisting of 387 males (62.6 %) and 231 females (37.4 %). Notably, the young age group (15–24 years), had the lowest frequency of hospitalized COVID-19 patients, while no case was observed in children (≤14 years) showing a significant association (p < 0.001) of age and disease prevalence. Comorbidities were observed in 63.9 % of COVID-19 patients including hypertension (HTN), diabetes mellitus (DM), ischemic heart diseases (IHD), asthma, chronic kidney disease (CKD) and tuberculosis (TB). The most common comorbidities were HTN (42.1 %) followed by DM (33.8 %), IHD (16.5 %), asthma (11.2 %), CKD (7.9 %) and TB (1.9 %).

Furthermore, the study revealed a significant association between comorbidities, age groups, and the need for non-invasive ventilation (NIV) (p < 0.001), mechanical ventilation (MV) (p < 0.001), and intensive care unit (ICU) admission (p < 0.001). Patients with specific comorbidities and those in the older age group (≥65 years) demonstrated a higher need for these interventions. However, patients without any comorbidity consistently exhibited the highest cumulative proportion of survival at each time point, indicating better overall survival outcomes. In contrast, patients with multimorbidities of DM/HTN/IHD, HTN/IHD, and DM/HTN/CKD had comparatively lower survival rates and higher mortality rates (p < 0.001).

**Conclusion:**

This research highlights the significant impact of age, comorbidities and multimorbidities on the severity and mortality of COVID-19 patients. It highlights the importance of considering these factors in tailoring effective management strategies for patients with COVID-19 or other infectious respiratory diseases.

## 1 Introduction

Infectious diseases have posed significant challenges to global health throughout history, causing devastating outbreaks and pandemics with immense human and economic costs. The emergence of the novel coronavirus disease 2019 (COVID-19) in late 2019 has further highlighted the need for a comprehensive understanding of the factors that contribute to disease severity and its subsequent impact on public health. Despite the development of various effective vaccines against the COVID-19 infection [[1], [2], [3], [4]], the associated risk factors are still not well understood and demand further research to combat with the disease.

The dynamic nature of the severe acute respiratory syndrome coronavirus 2 (SARS-CoV-2) and the continuous emergence of new variants urge to continuously investigate the factors influencing susceptibility, severity, and long-term consequences of the disease [5,6]. The SARS-CoV-2 primarily targets the respiratory system; however, other body organs effected with pre-existing comorbidities significantly influence disease outcomes [[7], [8], [9]]. Comorbidities are pre-existing medical conditions that can worsen the prognosis and increase the risk of complications in individuals infected with COVID-19 [10]. In developing countries like Pakistan, these risk factors are often overlooked due to healthcare resource constraints, impacting effective disease management.

Understanding the various risk factors associated with COVID-19 can aid in identifying highly susceptible cohorts and developing targeted public health interventions. It can also provide valuable insights into employing potential therapeutic strategies for these high-risk groups of patients. Therefore, relentless research efforts are essential to gain comprehensive knowledge of the disease, bolster preparedness, and mitigate future health threats, ensuring global health security beyond the current vaccination success.

This research paper aims to explore the effect of age and comorbidities on COVID-19 severity and mortality rate.

## 2 Material & methods

In this study, we employed a retrospective observational methodology to collect data from two main hospitals in Pakistan during the period from August 2021 to May 2022. These hospitals were declared as COVID isolation hospitals thereby representing patients from all over Pakistan including Isolation Hospital & Infectious Treatment Center (IHITC), Islamabad and Pakistan Institute of Medical Sciences (PIMS), Islamabad. The study was approved by the Institutional Review Board (IRB) of Quaid-i-Azam University, Islamabad, and ethical approval was obtained from the Ethical Review Committee of National Institute of Health, Islamabad. Throughout the research process, ethical considerations and patient confidentiality were diligently addressed. Written informed consent was obtained from all patients or their next of kin participating in the study. Personally identifiable information was removed during data collection and analysis to ensure compliance with data protection regulations.

To collect the required data, a thorough review of electronic medical records and personal files of patients was conducted to record the demographic information, such as age and gender. In addition, pre-existing comorbidities and multimorbidities, including hypertension (HTN), diabetes (DM), cardiovascular diseases (IHD), chronic kidney diseases (CKD), and respiratory illnesses such as Tuberculosis (TB) were documented as indicated in the medical records. This sample size ensured a minimum of 10 patients for each comorbidity, which was considered adequate for the retrospective analysis of the specific impact of comorbidities on COVID-19 outcomes.

Furthermore, the key disease outcomes, including the number of days of hospitalization, need for mechanical ventilation (MV), non-invasive ventilation (NIV), intensive care unit (ICU) admission, mortality and survival rate were recorded. Following data collection, the obtained information was entered into a structured electronic database/spreadsheet for subsequent analysis. The collected data was cross verified with patient records to ensure accuracy, consistency, and to minimize bias.

### 2.1 Statistical analysis

To ensure the integrity and reliability of the data analysis, incomplete records and cases without essential information were omitted. Descriptive statistics, such as frequencies, percentages, means, and standard deviations, were employed to summarize the demographic characteristics, prevalence of comorbidities, and mortality rate within the study sample. A chi-square test was performed to investigate the association between comorbidities and disease outcomes. Kaplan-Meier survival analysis was performed to find out the relationship between comorbidities/multi-morbidity on survival and death/mortality rate.

## 3 Results

### 3.1 Gender and age demographics of hospitalized COVID-19 patients

**Fig. 1:**
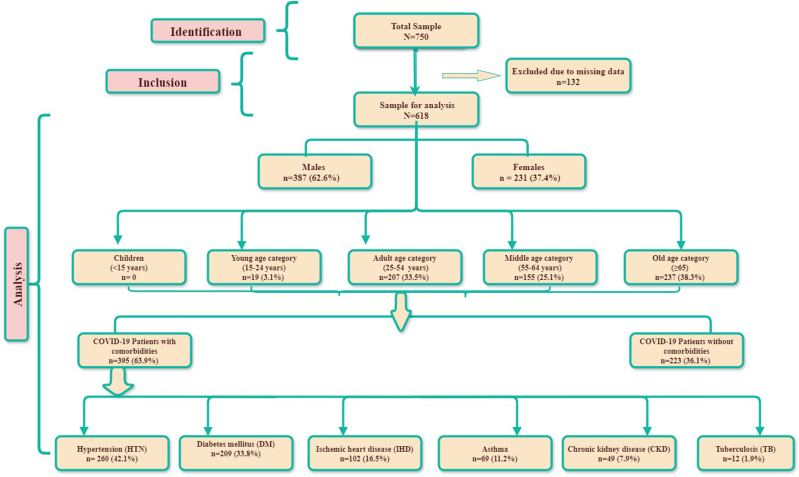
STROBE flow chart for retrospective observational study.

The present study examined a cohort of 618 out of 750 COVID-19 hospitalized patients. Data of 132 patients were excluded due to missing information and follow-up. The study cohort included 387 male (62.6 %) and 231 female (37.4 %) patients. Age distribution within the cohort revealed that the old age category (≥65) comprised the largest proportion, accounting for 237 (38.3 %) followed by the Adults (25–54 years) with 207 (33.5 %) patients and middle age (55–64 years) with 155 (25.1 %) patients. Notably, the young age category (15–24 years) displayed the lowest frequency, with a mere 19 (3.1 %) patients while no hospitalized COVID-19 patient was observed in children (≤14). The SRTOBE flow chart (Fig. 1) is depicting the inclusion criteria and number of samples for each category for the analysis.

### 3.2 Prevalence of comorbidities among hospitalized COVID-19 patients

In terms of comorbidity status, 223 (36.1 %) patients exhibited no underlying health conditions, while 395 (63.9 %) had comorbidities including Diabetes mellitus (DM), hypertension (HTN), asthma, ischemic heart disease (IHD), chronic kidney disease (CKD), and tuberculosis (TB) (Table 1).

**Table 1:** Prevalence of comorbidities in COVID-19 patients of different age groups.

Comorbidity	Total CM (%)	Age groups Young (15–24 years) Adult (25–54 years) Middle age (55–64 years) Old age (≥65 years)	No. of patients with comorbidity	Frequency in total COVID-19 patients (%)	Chi-Square test
No comorbidities	223 (36.1 %)	Young	18	2.9	χ^2^ = 101.395 df = 3 p = <0.001
		Adult	118	19.1	
		Middle age	39	6.3	
		Old age	48	7.8	
HTN	260 (42.1 %)	Young	0	0	χ^2^ = 44.836 df = 3 p <0.001
		Adult	60	9.7	
		Middle age	70	11.3	
		Old age	130	21	
DM	209 (33.8 %)	Young	0	0	χ^2^ = 26.332 df = 3 p <0.001
		Adult	51	8.3	
		Middle age	68	11	
		Old age	90	14.6	
IHD	102 (16.5 %)	Young	0	0	χ^2^ = 52.584 df = 3 p <0.001
		Adult	14	2.3	
		Middle age	17	2.8	
		Old age	71	11.5	
Asthma	69 (11.2 %)	Young	0	0	χ^2^ = 15.755 df = 3 p = 00.001
		Adult	11	1.8	
		Middle age	26	4.2	
		Old age	32	5.2	
CKD	49 (7.9 %)	Young	1	0.2	χ^2^ = 20.936 df = 3 p <0.001
		Adult	5	0.8	
		Middle age	10	1.6	
		Old age	33	5.3	
TB	12 (1.9 %)	Young	0	0	χ^2^ = 2.685 df = 3 p = 00.443
		Adult	2	0.3	
		Middle age	3	0.5	
		Old age	7	1.1	

**Abbreviations**: CM=Comorbidities, HTN= Hypertension, DM = Diabetes mellitus, IHD= Ischemic heart disease, CKD= Chronic kidney disease, TB = Tuberculosis.

Among the total sample population, HTN emerged as the most prevalent comorbidity, impacting a significant 42.1 % of individuals. DM followed closely, exhibiting a prevalence of 33.8 %. In contrast, other comorbidities such as asthma, IHD, CKD and TB demonstrated relatively lower prevalence at 11.2 %, 16.5 %, 7.9 %, and 1.9 % respectively. Interestingly, the prevalence of these comorbidities displayed variations across different age groups, adding intriguing nuances to the overall scenario.

Out of the total sample population (n = 618), 36.1 % did not have any comorbidity, with the highest proportion (19.1 %) belonging to the young age group (15–24 years). Interestingly, the absence of comorbidities showed a significant association with age groups (χ^2^ = 101.395, df = 3, p < 0.001), underscoring age-related differences in comorbidity occurrence. Remarkably, none of the patients in the young age group had hypertension, while the highest prevalence (21 %) was observed in the old age group (≥65 years). This notable disparity in hypertension prevalence across age groups indicated a significant association (χ^2^ = 44.836, df = 3, p < 0.001), hinting at potential age-specific risk factors. Similar to hypertension, none of the patients in the young age group had diabetes, while the highest prevalence was recorded in the old age group (14.6 %). Again, a statistically significant association between diabetes and age groups was established (χ^2^ = 26.332, df = 3, p < 0.001), highlighting the age-related impact of diabetes as a comorbidity in COVID-19.

The same age distribution pattern was observed in asthma, with no cases reported in the young age group. As age increased, the prevalence of asthma also increased, reaching its peak at 5.2 % in the old age group. The chi-square test confirmed a significant association between asthma and age groups (χ^2^ = 15.755, df = 3, p = 0.001), emphasizing the importance of considering asthma as a relevant factor in older COVID-19 patients.

Ischemic heart disease (IHD) was observed in 16.5 % of the total COVID-19 patients. Similar to hypertension and diabetes, none of the patients in the young age group had IHD. However, the prevalence of IHD increased substantially with age, with the highest occurrence in the old age group (11.5 %). The association between IHD and age groups was significant (χ^2^ = 52.584, df = 3, p < 0.001), highlighting its heightened relevance in elderly COVID-19 patients. Chronic kidney disease (CKD) was present in 7.9 % of the sample population, with only a small proportion in the young age group (0.2 %) and 5.3 % in the old age group showing a significant association between CKD and age groups (χ^2^ = 20.936, df = 3, p < 0.001).

Tuberculosis (TB) had the lowest frequency of 1.9 % in the total sample population of COVID-19 patients, with no cases reported in the young age group. The prevalence of TB as a comorbidity remained relatively low across all age groups, and no statistically significant association was observed between TB and age groups (χ^2^ = 2.685, df = 3, p = 0.443).

### 3.3 Association of comorbidities, age, and non-invasive ventilation (NIV) in COVID-19 patients

It was observed that patients without any comorbidity had a relatively low requirement for non-invasive ventilation (NIV), with only 2.9 % of the young age group requiring this intervention. However, as age increased, the need for NIV became more pronounced, with 19.1 %, 6.3 %, and 7.8 % of adult, middle age, and old age groups requiring this form of respiratory support, respectively (Table 2). This pattern suggests that the presence of comorbidities may contribute to an increased risk of respiratory distress requiring NIV, particularly among older individuals.

**Table 2:** Requirement of non-invasive ventilation (NIV) in COVID-19 patients of different age groups.

Comorbidity	Total patients with specific comorbidity (%)	Total patients receiving NIV with specific CM (%)	Age groups Young (15–24 years) Adult (25–54 years) Middle age (55–64 years) Old age (≥65 years)	No. of patients required NIV	Frequency (%)	Chi-Square test
No comorbidity	223 (36.1 %)	44 (19.7 %)	Young	3	1.3	χ^2^ = 30.472 df = 3 p <0.001
			Adult	10	4.5	
			Middle age	9	4	
			Old age	22	9.9	
HTN	260 (42.1 %)	97 (37.3 %)	Young	0	0	χ^2^ = 57.521 df = 3 p <0.001
			Adult	16	6.2	
			Middle age	28	10.8	
			Old age	53	20.4	
DM	209 (33.8 %)	93 (44.5 %)	Young	0	0	χ^2^ = 50.862 df = 3p <0.001
			Adult	19	9.1	
			Middle age	30	14.4	
			Old age	44	21.1	
IHD	102 (16.5 %)	55 (53.9 %)	Young	0	3.9	χ^2^ = 32.226 df = 3p <0.001
			Adult	4	6.9	
			Middle age	7	43.1	
			Old age	44	53.9	
Asthma	69 (11.2 %)	41 (59.4 %)	Young	0	0	χ^2^ = 45.018 df = 3 p <0.001
			Adult	6	8.7	
			Middle age	15	21.7	
			Old age	20	29	
CKD	49 (7.9 %)	27 (55.1 %)	Young	1	2	χ^2^ = 54.145 df = 3 p <0.001
			Adult	5	10.2	
			Middle age	6	12.2	
			Old age	15	30.6	
TB	12 (1.9 %)	10 (83.3 %)	Young	0	0	χ^2^ = 48.121 df = 3 p <0.001
			Adult	2	16.7	
			Middle age	3	25	
			Old age	5	41.7	

Abbreviations: CM=Comorbidities, HTN= Hypertension, DM = Diabetes mellitus, IHD= Ischemic heart disease, CKD= Chronic kidney disease, TB = Tuberculosis, NIV=Non-invasive ventilation.

Furthermore, when examining individual comorbidities, it was found that HTN, DM, IHD, and CKD showed a similar trend. Patients in the young age group with these comorbidities did not require NIV but its requirement increased with age. This highlights the importance of considering both age and specific comorbidities when assessing the likelihood of requiring NIV in COVID-19 patients.

In contrast, the comorbidities asthma and TB displayed different patterns. The need for non-invasive ventilation in COVID-19 patients with asthma was relatively low across all age groups, ranging from 0 % in the young age group to 5.2 % in the old age group. Similarly, patients with TB had a minimal requirement for non-invasive ventilation.

### 3.4 Association of comorbidities, age, and mechanical ventilation (MV) in COVID-19 patients

The need for mechanical ventilation (MV) varied based on both age and comorbidity among COVID-19 patients (Table 3). Patients without any comorbidity had a lower requirement for MV, while it increased as age and comorbidities increased. Specifically, in older age groups, a higher percentage of patients with HTN (14.6 %), IHD (32.4 %), and CKD (36.7 %) required MV. Likewise, TB and asthma showed a higher need for mechanical ventilation in older age groups. Statistical analysis using chi-square tests confirmed the significant associations between comorbidities, age groups, and the need for mechanical ventilation (p < 0.001). These findings underscore the importance of considering age and comorbidities in predicting the likelihood of requiring mechanical ventilation in COVID-19 patients.

**Table 3:** Requirement of mechanical ventilation (MV) in COVID-19 patients of different age groups.

Comorbidity	Total patients with specific comorbidity (%)	Total patients receiving MV with specific CM (%)	Age groups Young (15–24 years) Adult (25–54 years) Middle age (55–64 years) Old age (≥65 years)	No. of patients required MV	Frequency (%)	Chi-Square test
No comorbidity	223 (36.1 %)	17 (7.6 %)	Young	0	0	χ^2^ = 11.845 df = 3 p = 00.008
			Adult	5	2.2	
			Middle age	3	1.3	
			Old age	9	4	
HTN	260 (42.1 %)	63 (24.2 %)	Young	0	0	χ^2^ = 60.233 df = 3 p <0.001
			Adult	5	1.9	
			Middle age	20	7.7	
			Old age	38	14.6	
DM	209 (33.8 %)	52 (24.9 %)	Young	0	0	χ^2^ = 51.102 df = 3 p <0.001
			Adult	8	3.8	
			Middle age	12	5.7	
			Old age	32	15.3	
IHD	102 (16.5 %)	38 (37.3 %)	Young	0	0	χ^2^ = 38.774 df = 3 p <0.001
			Adult	2	2	
			Middle age	3	2.9	
			Old age	33	32.4	
Asthma	69 (11.2 %)	33(47.8 %)	Young	0	0	χ^2^ = 41.008 df = 3p <0.001
			Adult	1	1.4	
			Middle age	10	14.5	
			Old age	22	31.9	
CKD	49 (7.9 %)	21 (42.9 %)	Young	0	0	χ^2^ = 46.158 df = 3p <0.001
			Adult	1	2	
			Middle age	2	4.1	
			Old age	18	36.7	
TB	12 (1.9 %)	9 (75 %)	Young	0	0	χ^2^ = 53.122 df = 3 p <0.001
			Adult	0	0	
			Middle age	3	25	
			Old age	6	50	

Abbreviations: CM=Comorbidities, HTN= Hypertension, DM = Diabetes mellitus, IHD= Ischemic heart disease, CKD= Chronic kidney disease, TB = Tuberculosis, MV = Mechanical ventilation.

### 3.5 Association of comorbidities, age, and intensive care unit (ICU) admission in COVID-19 patients

COVID-19 Patients without any comorbidity had a lower requirement for ICU admission, whereas the presence of comorbidities increased the need for ICU admission. For example, in the old age group, 6.3 % of patients without comorbidities required ICU care, compared to 36.3 % of patients with IHD and 58.3 % of patients with TB (Table 4). When considering individual comorbidities, HTN, DM, asthma, IHD, CKD, and TB were associated with an increased need for ICU admission across different age groups. In older age groups, a higher percentage of patients with these comorbidities required ICU care.

**Table 4:** Requirement of ICU admission in COVID-19 patients of different age groups.

Comorbidity	Total patients with specific comorbidity (%)	Total patients in ICU with specific CM (%)	Age groups Young (15–24 years) Adult (25–54 years) Middle age (55–64 years) Old age (≥65 years)	No. of patients in ICU	Frequency (%)	Chi-Square test
No comorbidity	223 (36.1 %)	30 (13.5 %)	Young	3	1.3	χ^2^ = 14.866 df = 3 p = 00.002
			Adult	8	3.6	
			Middle age	5	2.2	
			Old age	14	6.3	
HTN	260 (42.1 %)	105 (40.4 %)	Young	0	0	χ^2^ = 39.655 df = 3 p <0.001
			Adult	8	3.1	
			Middle age	28	10.8	
			Old age	69	26.5	
DM	209 (33.8 %)	79 (37.8 %)	Young	0	0	χ^2^ = 61.994 df = 3 p <0.001
			Adult	9	4.3	
			Middle age	25	12	
			Old age	45	21.5	
IHD	102 (16.5 %)	43 (42.2 %)	Young	0	0	χ^2^ = 62.681 df = 3< p <0.001
			Adult	2	2	
			Middle age	4	3.9	
			Old age	37	36.3	
Asthma	69 (11.2 %)	34 (49.3 %)	Young	0	0	χ^2^ = 64.118 df = 3 p <0.001
			Adult	2	2.9	
			Middle age	10	14.5	
			Old age	22	31.9	
CKD	49 (7.9 %)	31 (63.3 %)	Young	0	0	χ^2^ = 55.681 df = 3p <0.001
			Adult	0	0	
			Middle age	4	8.2	
			Old age	27	55.1	
TB	12 (1.9 %)	10 (83.3)	Young	0	0	χ^2^ = 71.520 df = 3 p <0.001
			Adult	0	0	
			Middle age	3	25	
			Old age	7	58.3	

Abbreviations: CM=Comorbidities, HTN= Hypertension, DM = Diabetes mellitus, IHD= Ischemic heart disease, CKD= Chronic kidney disease, TB = Tuberculosis, ICU= Intensive care unit.

Chi-square tests confirmed the significant associations between comorbidities, age groups, and the need for ICU admission (p < 0.001) highlighting the importance of age and comorbidities to predict the likelihood of requiring intensive care and ICU admission in COVID-19 patients.

### 3.6 Association of comorbidities, age, and mortality rate

Patients without any comorbidity had a higher likelihood of recovery, with a lower percentage of death. On the contrary, the frequency of death/mortality is increased with burden of comorbidities, while the likelihood of recovery decreased (Table 5).

**Table 5:** Association of comorbidities, age, and mortality rate.

Comorbidity	Total patients with specific comorbidity (%)	Total deaths in COVID-19 patients with specific CM (%)	Age groups Young (15–24 years) Adult (25–54 years) Middle age (55–64 years) Old age (≥65 years)	No. of deaths	Frequency (%)	Chi-Square test
No comorbidities	223 (36.1 %)	9 (4 %)	Young	2	0.9	χ^2^ = 12.097 df = 3 p = 0.007
			Adult	4	1.8	
			Middle age	2	0.9	
			Old age	9	4	
HTN	260 (42.1 %)	97 (37.3 %)	Young	0	0	χ^2^ = 37.208 df = 3 p <0.001
			Adult	9	3.5	
			Middle age	22	8.5	
			Old age	66	25.4	
DM	209 (33.8 %)	73 (34.9 %)	Young	0	0	χ^2^ = 62.936 df = 3 p <0.001
			Adult	11	5.3	
			Middle age	19	9.1	
			Old age	43	20.6	
IHD	102 (16.5 %)	44 (43.1 %)	Young	0	0	χ^2^ = 57.696 df = 3 p <0.001
			Adult	4	3.9	
			Middle age	3	2.9	
			Old age	37	36.3	
Asthma	69 (11.2 %)	33 (47.8 %)	Young	0	0	χ^2^ = 57.569 df = 3 p <0.001
			Adult	1	1.4	
			Middle age	10	14.5	
			Old age	22	31.9	
CKD	49 (7.9 %)	27 (55.1 %)	Young	0	0	χ^2^ = 53.399 df = 3 p <0.001
			Adult	0	0	
			Middle age	3	6.1	
			Old age	24	49	
TB	12 (1.9 %)	10 (83.3 %)	Young	0	0	χ^2^ = 67.774 df = 3 p <0.001
			Adult	0	0	
			Middle age	3	25	
			Old age	7	58.3	

Abbreviations: CM=Comorbidities, HTN= Hypertension, DM = Diabetes mellitus, IHD= Ischemic heart disease, CKD= Chronic kidney disease, TB = Tuberculosis.

The comorbidity with the highest mortality rate was tuberculosis (TB), with 83.3 % of patients experiencing death. This was followed by chronic kidney disease (CKD) with a mortality rate of 55.1 %. Ischemic heart disease (IHD) had the next highest mortality rate at 43.1 %, followed by hypertension (HTN) at 37.3 %, diabetes mellitus (DM) at 34.9 %, and asthma at 47.8 %. Analyzing individual comorbidities, HTN, DM, asthma, IHD, CKD, and TB were associated with a higher frequency of death across different age groups. For example, in the old age group, patients with HTN had a higher frequency of death compared to those without HTN. Similar patterns were observed for other comorbidities, indicating an increased risk of death in patients with these underlying conditions.

### 3.7 Effect of multimorbidities on recovery and mortality rate in COVID-19 patients

**Fig. 2:**
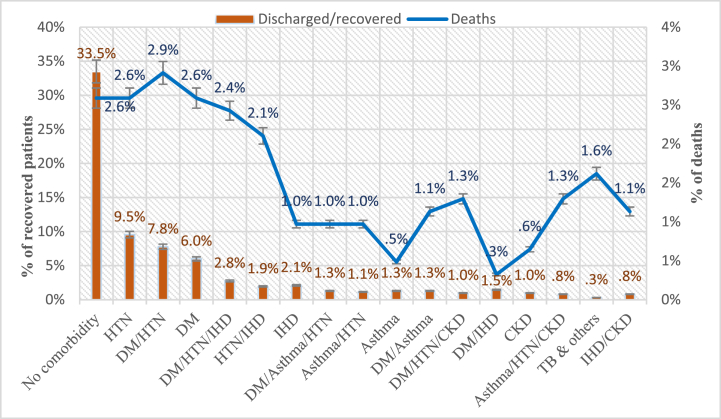
Frequency (%) of death and recovery in comorbid/multimorbid COVID-19 patients.

Out of a total of 618 COVID-19 patients, 457 (73.9 %) were discharged from hospital after recovery, while 161 (26.1 %) died. The data showed a clear association between the number of comorbidities and disease outcomes (p < 0.001). Among patients with no comorbidity, 207 (33.5 %) were recovered, and 16 (2.6 %) died. For patients with one comorbidity, 126 (20.4 %) were recovered, while 47 (7.6 %) died. Among those with two comorbidities, 88 (14.2 %) were recovered, and 58 (9.4 %) died. While patients with three or more comorbidities had 38 (6.1 %) recovered cases and 39 (6.3 %) deaths. The findings indicate a clear trend, with an increasing number of comorbidities correlating with a higher mortality rate among COVID-19 patients. The overall mortality rate with three and more comorbidities was slightly lower than (6.3 %) than with two comorbidities (9.4 %), possibly due to the combination of specific comorbidities based on their nature and severity. For example, individuals with multimorbidity of DM/HTN/IHD had a death rate of 2.4 %, the combination of DM/HTN/CKD resulted in a death rate of 1.3 %, and the combination of asthma/HTN/CKD showed a death rate of 1.3 % while patients with two comorbidities like DM/HTN showed the death rate of 2.9 % slightly higher than the previously mentioned 3 comorbidities (Fig. 2). Overall, these findings underscore the cumulative effect of multiple comorbidities, with a subsequent increase in the risk of adverse outcomes.

### 3.8 Kaplan-Meier survival analysis in COVID-19 patients

**Fig. 3:**
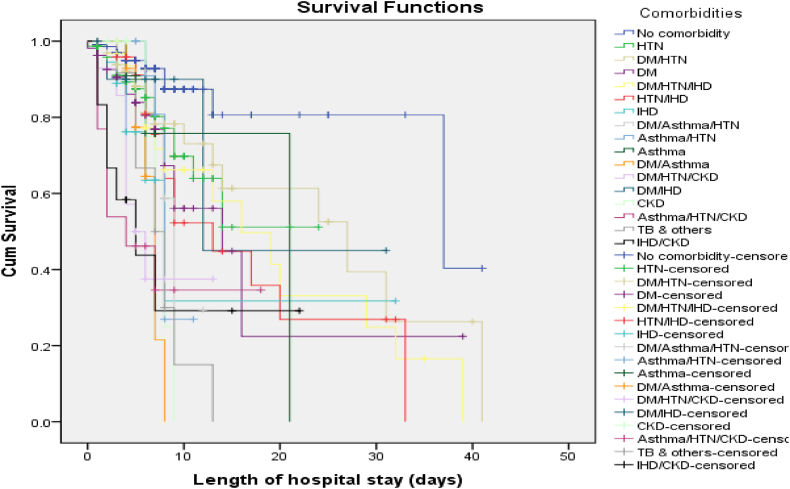
Association between comorbidities/multimorbidities and probability of survival.

The Kaplan-Meier survival analysis revealed distinct survival patterns based on the presence and combination of comorbidities. Patients without any comorbidity consistently exhibited the highest cumulative proportion of survival at each time point, indicating better overall survival outcomes (Fig. 3). In contrast, patients with multimorbidities of DM/HTN/IHD, HTN/IHD, and DM/HTN/CKD had comparatively lower survival rates.

The log-rank test was employed to determine the statistical significance of the observed differences in survival distributions among COVID-19 patients with different comorbidities/multimorbidities. The results indicated a significant association between comorbidity burden and survival outcomes in COVID-19 patients (p < 0.001) suggesting a notable impact on survival rate of patients.

Interestingly, an intriguing pattern emerged regarding the relationship between comorbidity burden and hospitalization duration. Patients with multimorbidities tended to have shorter hospital stays (mean duration: 8.5 days, 95 % CI: 7.2–9.9), while those with fewer comorbidities experienced longer hospitalization periods (mean duration: 14.6 days, 95 % CI: 12.9–16.2). Furthermore, patients with more comorbidities had a higher mortality rate (30 % at 30-day follow-up), indicating a greater risk of death. In contrast, patients with fewer comorbidities demonstrated a higher survival rate (80 % at 30-day follow-up) despite their longer hospital stays. It highlights the complex interplay between comorbidity burden, length of hospital stay, and survival rates in COVID-19 patients.

## 4 Discussion

The findings of our study on the prevalence of comorbidities among COVID-19 patients have significant implications for understanding the impact of underlying health conditions on disease outcomes also supported by previous studies on COVID-19 infection. In our study, Hypertension (42.1 %) and diabetes mellitus (33.8 %) were the most prevalent comorbidities with the most severe disease outcomes in the old age group of patients (≥65yr) while no case of hospitalized COVID-19 infection was observed in children.

In the literature, the overall occurrence of infections, disease severity and mortality rate is less in the children as compared to young (15–24 years), adults (25–54 years), middle age (55–64 years) and old age (≥65 years) groups. Several factors could justify this age distribution phenomenon. Children are less likely to have underlying health conditions or comorbidities that can worsen COVID-19 outcomes. Comorbidities such as hypertension, diabetes, and cardiovascular disease are more prevalent among adults and old aged COVID-19 patients. Furthermore, children express lower levels of the ACE2 receptor in their respiratory tracts, potentially reducing viral entry and replication. Children have more active and adaptable immune systems potentially aiding in viral control and mitigating severe inflammatory responses. Pre-existing immunity due to prior exposure to common cold-causing coronaviruses might offer partial protection, contributing to milder symptoms [11]. Additionally, the absence of common comorbidities in children might play a role in their generally less severe disease course [12]. Social behaviors, such as spending less time outdoors and reduced contact with high-risk individuals, could also influence COVID-19 prevalence and severity in children. Asymptomatic and mild infections might remain undetected, affecting the overall perception of severity and less hospitalization [13]. In addition to that, Vitamin D plays an important role in anti-inflammatory and antioxidative activities in the body. It gets deficient in the body with the passage of age and with underlying health conditions thus increasing the risk for the development of respiratory tract infections [14]. Despite milder symptoms, children can still transmit the virus, underscoring the need for continued preventive measures, including vaccination and public health interventions to protect vulnerable populations.

This study also shed light on the prevalence of certain comorbidities in the Pakistani population, which could contribute to the increased risk of severe COVID-19 outcomes. Based on the latest population-based National Health Survey of Pakistan (NHSP), it was determined that approximately 18.9 % of individuals aged above 15 years in Pakistan suffered from hypertension. The study highlighted a higher occurrence of hypertension in urban populations compared to rural areas, as well as a greater prevalence among men in contrast to women [15]. According to the most recent IDF Atlas data, Pakistan ranks third globally in terms of its substantial population affected by type 2 diabetes, with an estimated 33 million individuals impacted. Additionally, there are approximately 11 million adults in Pakistan affected with impaired glucose tolerance, and alarmingly, about 8.9 million people with diabetes remain undiagnosed [2,3]. Furthermore, asthma, IHD, CKD, and TB also exhibit considerable prevalence among the Pakistani population [16,17] thus acting as a significant risk factor in increasing the severity and mortality rate in COVID-19 patients [18].

These underlying conditions in COVID-19 patients can significantly compromise the body's ability to combat the SARS-CoV-2 virus. This weakened immunity may result in prolonged viral presence [19,20], delayed and less effective immune responses, impaired immune cell function [21], dysregulated inflammation leading to cytokine storms [22], and compromised immunological memory. Additionally, COVID-19 patients with comorbidities may be more susceptible to secondary infections, further complicating the disease course.

Recent studies by the COVID-19 Host Genetics Initiative have shown that genetic variations associated with comorbidities significantly impact COVID-19 severity. Several genetic loci, such as the TYK2 and DPP9 genes, have been identified that affect COVID-19 susceptibility and severity. Dysregulated TYK2 in diabetic patients is associated with signaling pathways involved in the immune response. Recent studies have shown that it is also associated with increased susceptibility to COVID-19 infection. The DPP9 gene, has also been found to be associated with diabetes mellitus, as well as the severity of COVID-19, thus justifying the increased disease severity in COVID-19 patients comorbid with diabetes [23,24]. Thus, the presence of comorbidities in COVID-19 patients significantly contributes to the severity of the disease through several mechanisms and underlying pathways [25] which require further investigation to combat COVID-19 and future infectious diseases.

It is important to acknowledge some limitations of this study. Being retrospective, these findings were dependent on the availability and completeness of medical records, which may have compromised the data quality to some extent. Moreover, the study's confinement to just two hospitals may limit the extent to which the findings can be applied to a broader population.

## 5 Conclusions

In conclusion, our research highlights the importance of understanding the impact of age and comorbidities on poor prognosis of COVID-19. This knowledge can help to identify the vulnerable cohort of patients and to design targeted interventions to improve disease management and reduce the burden of COVID-19 and other infectious diseases in healthcare systems. Furthermore, understanding the risk factors involved in disease susceptibility and severity can contribute to personalized approaches in managing and treating COVID-19 patients. This comprehensive understanding of risk factors and underlying conditions can lead to more effective public health strategies, better preparedness for future pandemics, and ultimately, improved global health outcomes.

## Funding

We extend our appreciation to the Researchers Supporting Project (No. RSP2024R436), at 10.13039/501100002383King Saud University, Riyadh, Saudi Arabia, for funding this study.

## Institutional review board statement

The study is approved by the institutional review board of Quaid-i-Azam University, Islamabad and Ethical review board of National Institute of Health, Islamabad. (Ethical Approval Reference No: F.1-5/RAPiD/2020–21/ERC).

## Informed consent statement

Written informed consent was obtained from all study subjects involved in the study.

## Availability of data

The data will be available from the corresponding author upon reasonable request. Restrictions apply to the availability of data due to privacy and ethical considerations.

## CRediT authorship contribution statement

**Maria Shoukat:** Writing – original draft, Software, Project administration, Methodology, Investigation, Formal analysis, Data curation, Conceptualization. **Haseeb Khan:** Methodology, Investigation, Data curation. **Wajid Munir:** Visualization, Resources, Investigation, Data curation. **Moona Nazish:** Writing – review & editing, Visualization, Resources. **Abdulwahed Fahad Alrefaei:** Writing – review & editing, Resources, Funding acquisition. **Mohammed Fahad Albeshr:** Writing – review & editing, Resources, Funding acquisition. **Anwar Ali:** Writing – review & editing, Resources, Funding acquisition. **Saad Ahmed:** Visualization, Validation, Data curation. **Afsheen Mansoor:** Writing – review & editing, Validation, Resources. **Massab Umair:** Writing – review & editing, Supervision, Methodology. **Muhammad Suleman Rana:** Writing – review & editing, Visualization. **Malik Badshah:** Writing – review & editing, Visualization, Supervision, Resources.

## Declaration of generative AI and AI-assisted technologies in the writing process

During the preparation of this work the authors used Chat GPT-4 to improve the writing skills. Authors carefully reviewed and edited the content as per requirements and take full responsibility for the content of the publication.

## Declaration of competing interest

The authors declare that they have no known competing financial interests or personal relationships that could have appeared to influence the work reported in this paper.
